# 17α‐estradiol acts through hypothalamic pro‐opiomelanocortin expressing neurons to reduce feeding behavior

**DOI:** 10.1111/acel.12703

**Published:** 2017-11-23

**Authors:** Frederik J. Steyn, Shyuan T. Ngo, Vicky Ping Chen, Lora C. Bailey‐Downs, Teresa Y. Xie, Martin Ghadami, Stephen Brimijoin, Willard M. Freeman, Marcelo Rubinstein, Malcolm J. Low, Michael B. Stout

**Affiliations:** ^1^ University of Queensland Centre for Clinical Research Faculty of Medicine Brisbane Qld Australia; ^2^ Department of Neurology Royal Brisbane & Women's Hospital Brisbane Qld Australia; ^3^ Wesley Medical Research Auchenflower Qld Australia; ^4^ Australian Institute for Bioengineering and Nanotechnology University of Queensland Brisbane Qld Australia; ^5^ Queensland Brain Institute University of Queensland Brisbane Qld Australia; ^6^ Robert and Arlene Kogod Center on Aging Mayo Clinic Rochester MN USA; ^7^ Department of Nutritional Sciences University of Oklahoma Health Sciences Center Oklahoma City OK USA; ^8^ School of Biomedical Sciences The University of Queensland Brisbane Qld Australia; ^9^ Department of Physiology University of Oklahoma Health Sciences Center Oklahoma City OK USA; ^10^ Reynolds Oklahoma Center on Aging University of Oklahoma Health Sciences Center Oklahoma City OK USA; ^11^ Harold Hamm Diabetes Center University of Oklahoma Health Sciences Center Oklahoma City OK USA; ^12^ Instituto de Investigaciones en Ingeniería Genética y Biología Molecular Consejo Nacional de Investigaciones Científicas y Técnicas Buenos Aires Argentina; ^13^ Facultad de Ciencias Exactas y Naturales Universidad de Buenos Aires Buenos Aires Argentina; ^14^ Department of Molecular and Integrative Physiology University of Michigan Medical School Ann Arbor MI USA

**Keywords:** 17α‐estradiol, aging, food intake, hypothalamus, obesity, pro‐opiomelanocortin

## Abstract

Weight loss is an effective intervention for diminishing disease burden in obese older adults. Pharmacological interventions that reduce food intake and thereby promote weight loss may offer effective strategies to reduce age‐related disease. We previously reported that 17α‐estradiol (17α‐E2) administration elicits beneficial effects on metabolism and inflammation in old male mice. These observations were associated with reduced calorie intake. Here, we demonstrate that 17α‐E2 acts through pro‐opiomelanocortin (*Pomc*) expression in the arcuate nucleus (ARC) to reduce food intake and body mass in mouse models of obesity. These results confirm that 17α‐E2 modulates appetite through selective interactions within hypothalamic anorexigenic pathways. Interestingly, some peripheral markers of metabolic homeostasis were also improved in animals with near complete loss of ARC *Pomc* transcription. This suggests that 17α‐E2 might have central and peripheral actions that can beneficially affect metabolism cooperatively or independently.

Weight loss through reduced energy intake curtails disease burden and metabolic perturbations associated with obesity in older age (Waters, Ward, & Villareal, 2013). However, reduced food intake and sustained weight loss are difficult to maintain in humans due to adverse effects with thermoregulation, libido, satiety, and musculoskeletal mass (Dirks & Leeuwenburgh, 2006). These compliance issues have promoted research interest into pharmacological interventions that promote reductions in food intake without having to voluntarily restrict dietary intake. We recently reported that 17α‐estradiol (17α‐E2), a naturally occurring enantiomer of 17β‐estradiol, produces beneficial effects on metabolism and inflammation in old male mice (Stout et al., 2017). These effects may contribute to the reported extension of lifespan by 17α‐E2 (Harrison et al., 2014; Strong et al., 2016), which may result from central and peripheral effects on food intake and nutrient‐sensing pathways (Stout et al., 2017). Here, we demonstrate that 17α‐E2 promotes weight loss in male mouse models of obesity, showing that the beneficial effects of 17α‐E2 on food intake and body weight require a functional threshold level of *Pomc* expression in the hypothalamic arcuate nucleus (ARC).

We first assessed the effect of dietary 17α‐E2 treatment on body mass and composition, food intake, spontaneous activity, and energy expenditure in male mice maintained on an obesogenic diet. 17α‐E2 quickly initiated weight loss (Figure 1a), resulting in a significant decrease in body mass at the end of the study (Figure 1b). The reduction in body mass was observed despite continued high‐fat feeding and was attributed to significant declines in fat mass (Figure 1c–d), sparing lean mass as we previously reported (Stout et al., 2017). We also observed significantly enhanced glucose tolerance, evidenced by increased glucose disposal and decreased insulin secretion during an intraperitoneal glucose challenge (Figure 1e) and reductions in fasting glucose and insulin levels (Figure 1f). We performed a phenotypic assessment during week 20 of the intervention to determine the cause of weight reduction. 17α‐E2 reduced food intake during the week of assessment, with the majority of these effects occurring during the dark cycle (Figure 1g–j). 17α‐E2 did not reverse HFD‐mediated reductions in locomotor activity (Figure 1k–m), nor did it alter metabolic rate (Figure 1n–o), suggesting that 17α‐E2‐mediated effects on body mass and composition are driven by changes in food intake. Isolation and placement of mice into metabolic cages could potentially alter energy balance; therefore, changes in energy expenditure with 17α‐E2 cannot be completely excluded. To show that changes in food intake did not result from poor diet palatability, we evaluated body mass, body composition, and food intake in mice treated with subcutaneous slow‐release 17α‐E2 pellets. As with dietary treatment, subcutaneous 17α‐E2 treatment initiated dose‐dependent declines in body mass (Figure 1p), adiposity (Figure 1q), and energy intake (Figure 1r). We subsequently focused on unraveling mechanisms through which 17α‐E2 modulates feeding behavior.

**Figure 1:**
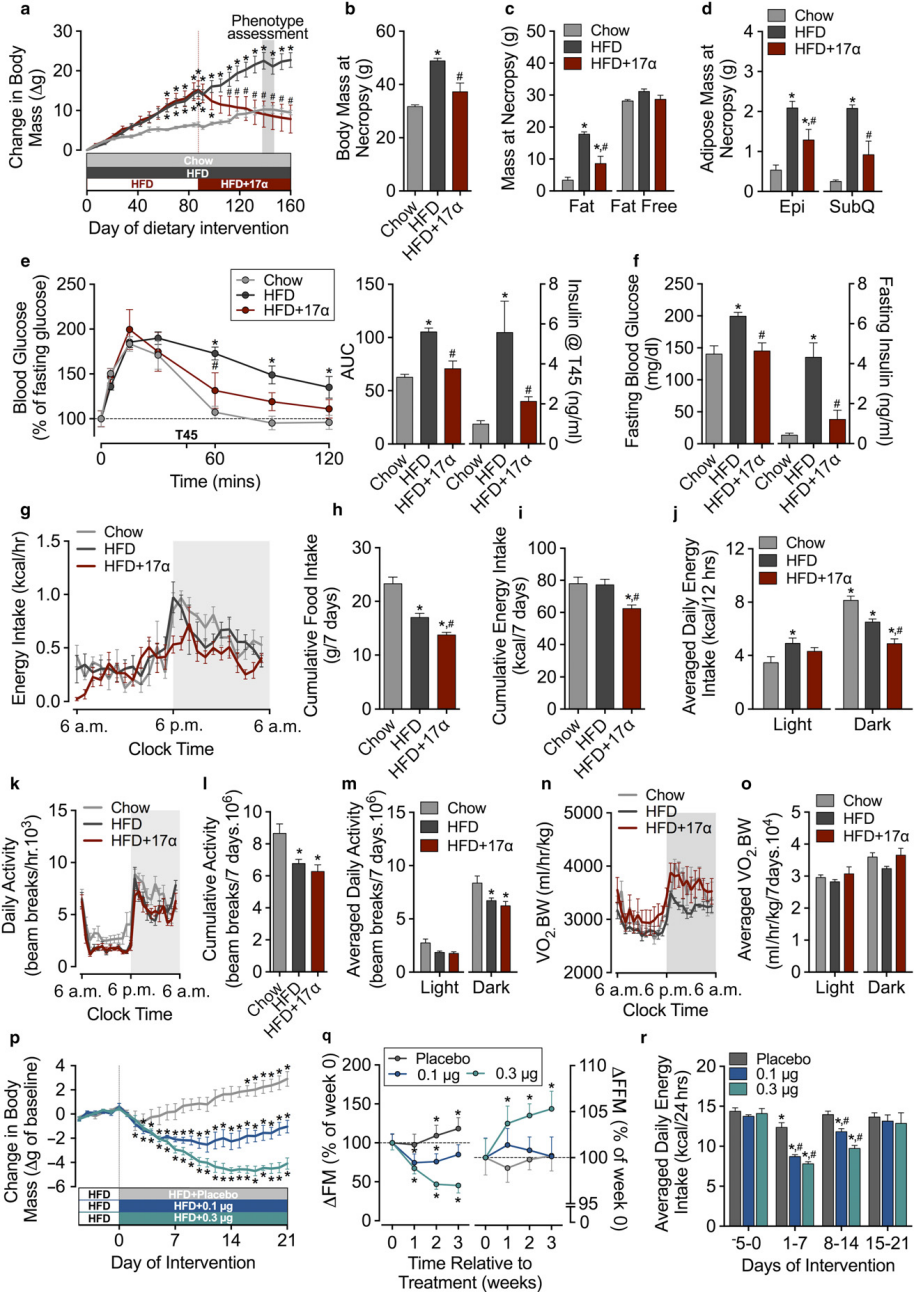
17α‐E2 reverses high‐fat diet (HFD)‐mediated perturbations in adiposity and metabolism by reducing dietary intake. (a) Change in body mass in mice fed chow, HFD, or HFD switched to HFD+17α‐E2. (b) Body mass, (c) fat and fat‐free mass, and (d) epididymal (Epi) and inguinal (SubQ) adipose mass at necropsy. (e) Normalized blood glucose, area under the curve (AUC), and blood insulin levels during intraperitoneal glucose tolerance testing (IP‐GTT) during week 23 of the study. (f) Fasting blood glucose and insulin prior to IP‐GTT. Phenotypic measures collected during week 20 of the study, including (g) energy intake over a representative 24‐hour sampling period, (h) cumulative weekly food, and (i) energy intake, (j) average daily energy intake during light and dark periods, (k) daily activity over a representative 24‐hour sampling period, (l) cumulative weekly activity, (m) averaged daily activity during light and dark periods, (n) oxygen consumption (VO_2_) normalized to body mass over a representative 24‐hour sampling period, and (o) averaged VO_2_ normalized to body mass over the 7‐day assessment period during light and dark periods. Change in (p) body mass, (q) body composition, and (r) averaged daily energy intake in mice implanted with subcutaneous cholesterol matrix pellets releasing either 0.0 (placebo), 0.1, or 0.3 μg/day 17α‐E2. All data are expressed as mean ± SEM (A‐0: *N *= 6/group; P‐R: *N *= 5/group). For A‐O, *p *< .05 considered statistically different from chow (*) or HFD (^#^) treated mice. For P‐Q, *p* < .05 from baseline (*). For R, *p* < .05 from baseline (*, days −5 to 0), or placebo (^#^) during respective treatment periods

We and others have previously reported that 17α‐E2 reduces food intake by acting through hypothalamic pathways (Butera, Beikirch, & Willard, 1990; Stout et al., 2017). *Pomc*‐expressing neurons located within the ARC constitute the dominant anorexigenic node of appetite regulating neurons and are viewed as key regulators of energy homeostasis. Activation of these neurons via peripheral appetite regulators such as leptin (Cowley et al., 2001) and insulin (Benoit et al., 2002) promotes satiety and diminishes food intake. Given our previous observation that 17α‐E2 treatment increased hypothalamic transcripts of the melanocortin system (Stout et al., 2017), we reasoned that 17α‐E2 might promote satiety in HFD fed mice through *Pomc*‐expressing neurons. To test this, we investigated the effects of dietary 17α‐E2 administration on food intake in mutant strains of mice with selectively reduced or nearly eliminated constitutive ARC *Pomc* expression.

Cooperative interactions between two POMC‐neuron‐specific enhancers, nPE1 and nPE2, promote expression of *Pomc* transcripts in the mouse ventromedial hypothalamus (Franchini et al., 2011; de Souza et al., 2005). Deletion of nPE2, nPE1, or insertion of a transcription‐blocking *neo* selection cassette into the vicinity of the two hypothalamic neuronal *Pomc* enhancers reduces hypothalamic *Pomc* expression to ~80%, ~30%, or ~2% of wild‐type controls, respectively (Lam et al., 2015). A reduction in hypothalamic *Pomc* expression at or below ~30% of wild‐type controls in these mice results in a functional loss of *Pomc*‐mediated regulation of body mass (Bumaschny et al., 2012; Lam et al., 2015; Zhan et al., 2013). Therefore, we hypothesized that if 17α‐E2 were to act selectively by increasing hypothalamic *Pomc* expression, the treatment effects on body mass and food intake would be disrupted in mutant mice lacking nPE1 (*Pomc* ^*Δ1*^) or those containing the *Pomc* transcription‐blocking *neo* selection cassette (*Pomc* ^*neo*^). Similar to experiments in Study 1, mice with loss of nPE1, loss of nPE2 (*Pomc* ^*Δ2*^), *neo* insertion into the *Pomc* gene, and their wild‐type sibling controls were treated with HFD containing 17α‐E2 in Study 3.

17α‐E2 treatment during high‐fat feeding immediately initiated weight loss in WT control mice (*Pomc* ^*wt*^; Figure 2a), promoting a near 20% reduction in body mass by week 3 of treatment (Figure 2b). Similar treatment effects were observed in *Pomc* ^*Δ2*^ mice, which maintain functional POMC activity (Lam et al., 2015) despite a ~40% reduction in constitutive ARC *Pomc* expression at the time of necropsy (Figure 2c). In *Pomc* ^*wt*^ and *Pomc* ^*Δ2*^, this change in body mass occurred in conjunction with an immediate decline in food intake (Figure 2d) that was followed by a slow rebound in energy intake by week 3 (Figure 2e). In contrast, *Pomc* ^*Δ1*^ and *Pomc* ^*neo*^ mice showed no 17α‐E2 treatment effects on body mass or food intake (Figure 2a–b, d–f). In *Pomc* ^*wt*^ and *Pomc* ^*Δ2*^ mice, the loss in body mass following 17α‐E2 administration was primarily attributed to loss of fat mass (Figure 2g–h). Supporting our hypothesis that the lack of treatment effects of 17α‐E2 in *Pomc* ^*Δ1*^ and *Pomc* ^*neo*^ mice was due to the lack of sufficient hypothalamic *Pomc* expression, measurement of ARC *Pomc* mRNA levels confirmed very low *Pomc* expression (Figure 2c), despite 3 weeks of 17α‐E2 treatment. These observations demonstrate that 17α‐E2 promotes satiety and reduces food intake, thereby inducing weight loss and reducing adiposity through functional hypothalamic *Pomc* gene transcription.

**Figure 2:**
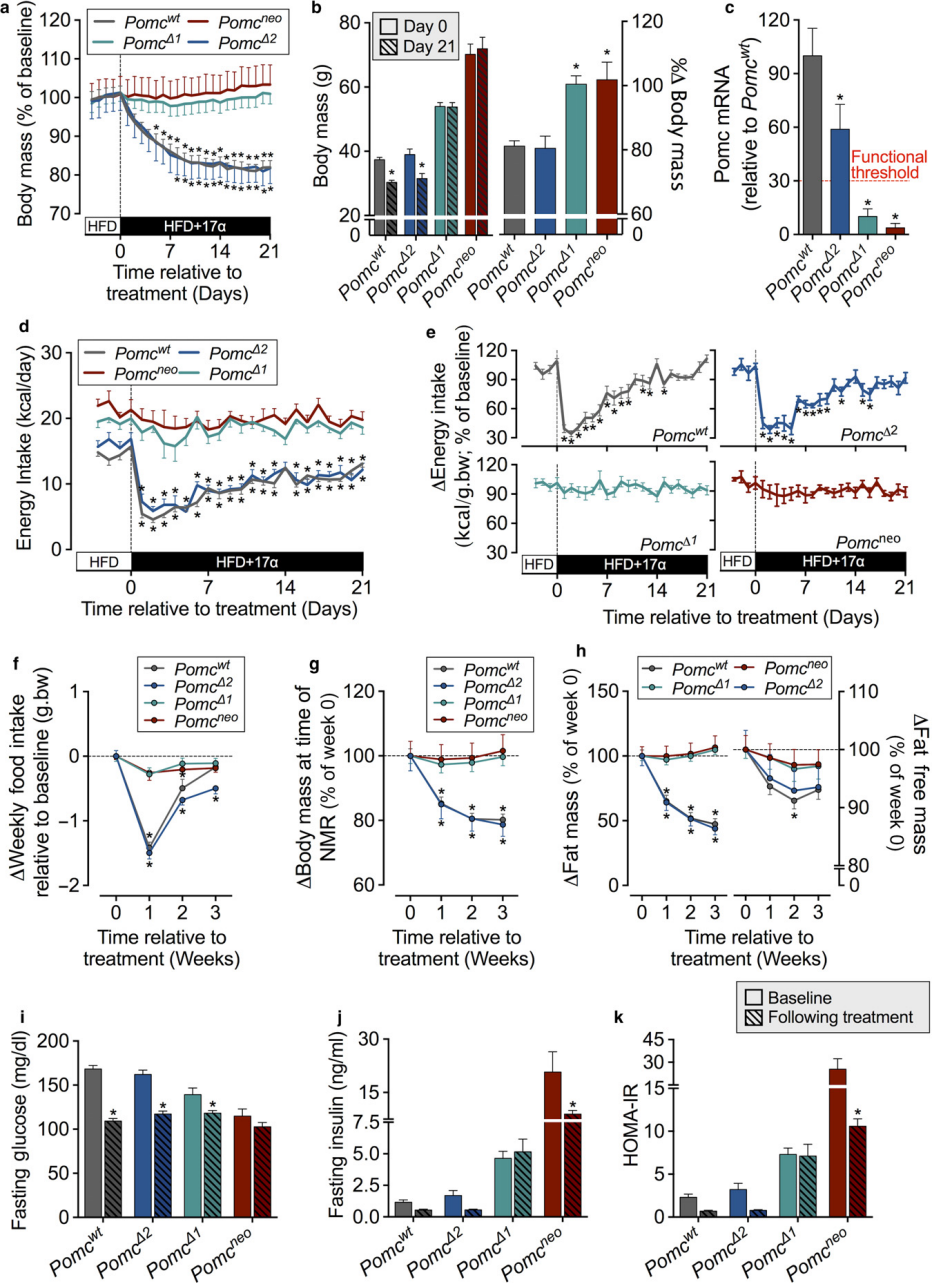
17α‐E2‐mediated effects on food intake, body mass, and adiposity are dependent upon hypothalamic *Pomc* gene transcription. (a) Change in body mass, normalized to baseline, following administration of 17α‐E2. (b) Actual (left) and percent change (right) in body mass relative to baseline at necropsy. (c) Hypothalamic *Pomc* expression in *Pomc* ^*wt*^ *, Pomc* ^*Δ2*^ *, Pomc* ^*Δ1*^, and *Pomc* ^*neo*^ mice at necropsy. (d) Daily energy intake before and following administration of 17α‐E2. (e) Percent change in energy intake, normalized to body mass, before and following 17α‐E2 treatment. Weekly (f) food intake, (g) body mass, and (h) fat and fat‐free mass, normalized to baseline, following 17α‐E2 treatment. (i) Fasting glucose, (j) fasting insulin, and (k) homeostatic model assessment of insulin resistance (HOMA‐IR) at baseline and week 3 of the study following administration of 17α‐E2. All data are expressed as mean ± SEM (Pomc^WT^ *N *= 12; Pomc^Δ2^ *N *= 7; Pomc^Δ1^ *N *= 9; Pomc^neo^ *N *= 7) with *p* < .05 considered statistically different from baseline (*; panels a–b,d–k) or *Pomc* ^*wt*^ (*; panels b–c)

To determine whether 17α‐E2‐mediated effects on food intake, body mass, and adiposity also modulate metabolic homeostasis, we assessed fasting glucose and insulin at baseline and week 3 of treatment. In alignment with Study 1, 17α‐E2 treatment decreased fasting glucose in *Pomc* ^*wt*^, *Pomc* ^*Δ2*^, and *Pomc* ^*Δ1*^ mice (Figure 2i). There was no change in fasting glucose levels in *Pomc* ^*neo*^ mice, but as previously demonstrated, these mice are resistant to developing hyperglycemia because of a lower renal threshold for glycosuria (Chhabra et al., 2016). Interestingly, 17α‐E2 lowered fasting insulin in *Pomc* ^*neo*^ mice (Figure 2j), an effect mirrored in the HOMA‐IR data (Figure 2k). The physiological relevance of this modest reduction remains unclear. Collectively, these data suggest that metabolic improvements by 17α‐E2 may not be solely driven by declines in food intake and body mass. Future studies are needed to definitively determine whether 17α‐E2 acts independently of ARC *Pomc* transcripts to improve systemic metabolic parameters.

We conclude that 17α‐E2 acts via hypothalamic *Pomc* transcripts to reduce food intake, thereby promoting reductions in body mass and adiposity in male mouse models of obesity. By isolating the central effects of 17α‐E2 to ARC *Pomc*, we gained insight into the mechanisms of 17α‐E2 actions and established the basis for future experiments to explore beneficial effects of 17α‐E2 that may occur independent of central appetite regulation.

## CONFLICT OF INTEREST

None declared.

## AUTHOR CONTRIBUTIONS

F.J.S. and M.B.S. conceived the project and designed the experiments. F.J.S. and M.B.S. performed the experiments with contributions from S.T.N., V.P.C., L.C.B., S.B., W.M.F., T.Y.X., and M.G. M.J.L. and M.R. provided mice and technical support related to data analysis and edited the manuscript. F.J.S. and M.B.S. wrote the manuscript and completed all revisions. All authors edited and approved the final manuscript.
